# Swedish Spring Wheat Varieties with the Rare High Grain Protein Allele of *NAM-B1* Differ in Leaf Senescence and Grain Mineral Content

**DOI:** 10.1371/journal.pone.0059704

**Published:** 2013-03-21

**Authors:** Linnéa Asplund, Göran Bergkvist, Matti W. Leino, Anna Westerbergh, Martin Weih

**Affiliations:** 1 Department of Crop Production Ecology, Swedish University of Agricultural Sciences, Uppsala, Sweden; 2 Swedish Museum of Cultural History, Julita, Sweden; 3 IFM - Biology, Linköping University, Linköping, Sweden; 4 Department of Plant Biology and Forest Genetics, BioCenter, Swedish University of Agricultural Sciences, Uppsala, Sweden; University College London, United Kingdom

## Abstract

Some Swedish spring wheat varieties have recently been shown to carry a rare wildtype (wt) allele of the gene *NAM-B1*, known to affect leaf senescence and nutrient retranslocation to the grain. The wt allele is believed to increase grain protein concentration and has attracted interest from breeders since it could contribute to higher grain quality and more nitrogen-efficient varieties. This study investigated whether Swedish varieties with the wt allele differ from varieties with one of the more common, non-functional alleles in order to examine the effect of the gene in a wide genetic background, and possibly explain why the allele has been retained in Swedish varieties. Forty varieties of spring wheat differing in *NAM-B1* allele type were cultivated under controlled conditions. Senescence was monitored and grains were harvested and analyzed for mineral nutrient concentration. Varieties with the wt allele reached anthesis earlier and completed senescence faster than varieties with the non-functional allele. The wt varieties also had more ears, lighter grains and higher yields of P and K. Contrary to previous information on effects of the wt allele, our wt varieties did not have increased grain N concentration or grain N yield. In addition, temporal studies showed that straw length has decreased but grain N yield has remained unaffected over a century of Swedish spring wheat breeding. The faster development of wt varieties supports the hypothesis of *NAM-B1* being preserved in Fennoscandia, with its short growing season, because of accelerated development conferred by the *NAM-B1* wt allele. Although the possible effects of other gene actions were impossible to distinguish, the genetic resource of Fennoscandian spring wheats with the wt *NAM-B1* allele is interesting to investigate further for breeding purposes.

## Introduction

Knowledge on the efficiency of agricultural crops in using N and other nutrients can help reduce e.g., nitrogen (N) fertilization and increase the value of the crops produced. There are many factors which affect nitrogen use, ranging from field management to soil, weather, and genotype of the crop, and their interactions [1]. For example, the genotypic traits affecting N uptake, N conversion to harvested product, and N retranslocation into plant parts that survive until the next growing season (e.g., grain) all influence N use efficiency [2]. Breeding could therefore be one method for improving N use efficiency.

Effective translocation of nitrogen to the grain during grain filling has been identified as a candidate trait for improving N use efficiency in bread wheat (*Triticum aestivum* L. ssp *aestivum*) [3], [4]. One gene possibly involved in nutrient translocation, and thereby nitrogen use efficiency, in wheat is *NAM-B1 (Gpc-B1)*. The locus on chromosome 6B was originally identified in tetraploid wild emmer wheat (*T. turgidum* L. ssp. *dicoccoides*) using durum wheat (*T. turgidum* L. ssp. *durum*) - wild emmer wheat substitution lines [5], [6] and later the gene has been mapped more precisely [7]. The effects of the locus on grain protein concentration have been shown in several tetraploid wheat backgrounds [8]–[10] and in hexaploid wheats [10]–[13]. The wildtype (wt) allele of *NAM-B1* originating in emmer wheat codes for a NAC-domain protein, a group of proteins known to be transcription factors involved in plant development processes. There are at least two additional alleles of the gene, both of which are believed to be non-functional. One has a +1 bp insertion likely causing a frame shift and a loss of the NAC-domain, the other one probably has a large deletion [14], [15].

Studies indicate that the functional wt *NAM-B1* allele increases the rate of senescence and allows more effective translocation of nutrients to the grain, resulting in shorter grain filling and higher concentrations of protein, Fe, Zn, and Mn in the grain [10], [14], [16]–[18]. The hexaploid wheat genome has several *NAM* homologues, which have been down-regulated with RNAi in a hexaploid line (carrying a non-functional *NAM-B1* allele). At 12 days after anthesis there are changes in the expression pattern of 691 genes [19], indicating involvement of *NAM* genes in complex mechanisms governing the senescence of leaves and the associated retranslocation of nutrients. Wildtype varieties have down-regulation of genes likely related to functions no longer needed during senescence, such as signaling components and photosynthetic machinery components, and up-regulation of genes possibly involved in the onset of senescence, such as proteins induced by the hormones jasmonic acid and abscissic acid. Effects of the wt allele on weight per grain and grain yield differ with genetic background and environment. When six hexaploid and three tetraploid near isogenic lines (NILs) with and without the wt allele were grown in the field, the wt allele had a negative effect on weight per grain and no significant effect on grain yield [12]. Mainly negative but sometimes positive effects on weight per grain in different genotype×environment combinations of three tetraploid recombinant substitution lines have also been reported [10]. In the previously mentioned RNAi line, grain protein concentration decreased but there was no significant change in weight per grain in a greenhouse experiment [14].

It has been hypothesized that the reduction in weight per grain in the wt genotype in many environments led to fixation of the non-functional alleles in durum and bread wheat during domestication [20]. However, the wt allele was found in four hexaploid wheat varieties (two spelt wheats, one spring and one winter bread wheat) out of 63 wheat varieties in a museum collection of wheat varieties from 1865 [21]. It was subsequently found in five (four spring wheats and one winter wheat) out of 367 varieties in a core collection of bread wheat chosen to maximize the world’s collected genetic diversity in bread wheat [15], [22]. Since many of the wt varieties in the museum and in the core collection had a northern origin and were spring wheats, a larger set of 138 northern spring wheat cultivars was screened. The wt allele was found only in Fennoscandian varieties, where 46 out of 104 varieties investigated carried the wt allele [15]. To our knowledge, the possible effects of the wt allele in Fennoscandian varieties have not previously been studied.

Besides yield, fast maturation and good baking quality have been important traits in Swedish spring wheat breeding [23]. Both these traits are positively affected by the wt *NAM-B1* allele [14], [24]. Even though the presence of the wt allele has declined during 20^th^ century breeding [15], these positive influences could explain the preservation of the allele during breeding despite its possible negative influence on yield.

Although effects of *NAM-B1* have been identified in NILs, these effects are not necessarily so large and consistent in different genotypic backgrounds that they would be visible when studying the allele in diverse varieties. The recently identified Swedish varieties with the wt allele therefore offer a chance to investigate whether varieties with the wt allele of *NAM-B1* show a different phenotype than varieties with a non-functional allele when present in different genetic backgrounds. In the present pot experiment, we compared flag leaf senescence, yield, weight per grain, and nutrient content in a set of Swedish varieties with and without the wt allele under controlled conditions in a climate chamber. Our starting hypothesis was that varieties with the wt allele have faster senescence and higher concentrations of the minerals N, Fe, Zn, and Mn in the grain than varieties with a non-functional allele. We also sought to explain why the wt allele is relatively common in Swedish varieties and to give an understanding of how modern breeding has affected N-retranslocation and senescence. Such information could be useful for breeders interested in possible effects of the wt allele of *NAM-B1* in different genetic backgrounds.

## Materials and Methods

### Plant Material

Seeds of 41 varieties of spring wheat were used (Table 1), of which 38 were donated by NordGen and three by a local farmer. Forty of the varieties were hexaploid spring wheat (*Triticum aestivum* L. ssp *aestivum*) and one was tetraploid emmer wheat (*T. turgidum* L. ssp. *dicoccoides*), which was included for comparison. The selection included three landraces with uncertain background cultivated for 10 years by a farmer in Uppsala, Sweden, six landraces with Swedish origin preserved in NordGen, and 29 Swedish cultivars released during the 20^th^ century. Three varieties from other countries which were used in early Swedish breeding were also included. Some measurements were only performed on a subset of 12 varieties with release years spanning the time period of all varieties (Table 1). All varieties were genotyped as described in [15]. The wt allele was present in 12 of the varieties, and the deletion allele (del) in 29 varieties. In the subset of 12 varieties, five had the wt allele.

**Table 1:** Varieties used in the study, their allele types and year of release, with the subset of 12 varieties indicated in bold style.

Deletion (non-functional) allele of *NAM-B1*			Wildtype (functional) allele of *NAM-B1*		
Acc. No.	Name	Year of release	Acc. No.	Name	Year of release
**NGB6675**	**Vårpärl**	**1901**	**NGB6678**	**Rubin**	**1921**
**NGB6676**	**Kolben**	**1892**	**NGB6679**	**Diamant**	**1928**
NGB6677	Extra Kolben	1919	**NGB6684**	**Rival**	**1952**
NGB6680	Fylgia I	1933	**NGB6688**	**Prins**	**1962**
**NGB6682**	**Progress**	**1941**	NGB6689	Amy	1971
NGB6683	Ella	1950	NGB13346	Sopu (Finland)	1935
NGB6685	Fylgia II	1952	NGB4499	Emmer wheat from Gotland **	landrace
**NGB6686**	**Drott**	**1955**	NGB6409	Halland	landrace
NGB6687	Safir	1955	**NGB6410**	**Dalarna**	**landrace**
NGB7455	Atle	1936	NGB6673	Landrace from Dalarna	landrace
NGB7456	Brons	1945	NGB13441	Västergötland	landrace
NGB7457	Kärn	1946	*	origin Öland	landrace
NGB7461	Svenno	1953			
NGB7462	Ring	1957			
NGB7464	Pompe	1967			
NGB7465	Snabbe	1968			
NGB7475	Kadett	1981			
NGB7479	Tjalve	1990			
NGB8923	Extra Kolben II	1926			
NGB9691	Blanka	1950			
**NGB9954**	**Dragon**	**1988**			
**NGB9955**	**Dacke**	**1990**			
NGB9956	Sport	1991			
NGB11010	Heines Kolben (Germany)	1900***			
NGB11280	Marquis (Canada)	1909			
**NGB13917**	**Vinjett**	**1998**			
NGB9708	Dalarna	landrace			
*	origin Dalarna	landrace			
*	origin Halland	landrace			

* Landrace obtained from farmer, all other accessions were obtained from NordGen.

** *Triticum turgidum* ssp. *dicoccon,* was not included in the comparison of allele types.

*** Exact year of origin unclear, 1900 was used in calculations.

### Growth Conditions

The experiment was laid out in a complete randomized block design with four replicates and with pot as the experimental unit. The experiment was conducted in a climate chamber with 16 h light, a 9/18°C night/day temperature regime, PAR about 230 µmol m^−2^ s^−1^ at the top of the pots, and 60% relative humidity. The pots were 13 cm×13 cm×13 cm in size, with a volume of 1.5 L, and were filled with a fertilized soil mixture containing (% of volume) 72% peat, 20% perlite, 5.6% silica clay, and 2.4% gravel. The soil initially contained 260 mg N per pot and essential macro- and micronutrients (g m^−3^, N 180, P 110, K 195, Mg 260, S 100, Ca 2000, Fe 6.0, Mn 3.5, Cu 2.5, Zn 1.5, B 0.6, Mo 3.0). Five seeds were sown per pot at a depth of 2.5 cm. Most of the plants had emerged after seven days, and day seven after sowing was considered day 1 of the experiment. The plants were thinned down to two plants per pot within the following week.

The pots were placed on trolleys, with about nine pots per trolley. The trolleys were moved within the blocks three times per week and the order of the blocks was rotated once a week until day 64, when moving the plants became impractical. The pots were initially placed on nets but were moved to trays on day 13 to allow nutrient solution to be soaked up when added later on. Nets were mounted above the trolleys to provide support when the plants grew taller. On day 69 the plants were moved to lower trolleys to allow further vertical growth of the plants. The plants were irrigated on the trays, initially only with deionized water, but from day 27 to 122 the plants were also supplied two times a week with 0.2 L nutrient solution per pot containing (mg L^−1^) 102 N, 20 P, 86 K, 8 S, 6 Ca, 8 Mg, 0.34 Fe, 0.4 Mn, 0.2 B, 0.06 Zn, 0.03 Cu, 0.0008 Mo. From day 123 the dose was reduced to 0.1 L nutrient solution applied twice a week. The plants were watered with deionized water as needed to keep the soil moist, with up to two days between watering occasions. The beginning of anthesis of the main stem in each pot (BBCH 61, [25]) was recorded as the day when one of the two plants had reached this stage.

### Measurements

Leaf chlorophyll content (SPAD index) was assessed with a portable chlorophyll meter (SPAD-502, Konica Minolta Sensing Inc., Japan). Two values were taken per pot, from the newest fully developed leaf on the main shoot of both plants. The value for each leaf was the mean of three measurements taken from along the middle of the leaves. Starting from a few days before anthesis, SPAD measurements of the main stem flag leaf of all plants were taken initially three, then two times a week. Each of the two main shoots in each pot was individually labeled, using two different colors of cotton thread. When it was no longer possible to get a value (on dying leaves), it was recorded as zero.

The harvest was performed block-wise on days 138 and 139, when all plants were considered completely mature by visual inspection. At harvest, the number of ears was counted in each pot and the height was measured from the soil surface to the tip of the ears, excluding awns on the two main stems in each pot. The ears were cut off and stored at room temperature in open paper bags. The remaining straw was cut at ground level and dried for 48 hours at 60°C. The ears were threshed by hand and the grains and chaff were thereafter dried. The number of grains was counted by hand in each sample and weight per grain was calculated by dividing the dry weight by the number of grains. Straw (including chaff) and half of each grain sample were milled using a knife mill (Grindomix GM 200, Retsch GmbH) with titanium blade to minimize micronutrient contamination [26]. All grain samples and a subset (12 varieties) of straw samples were analyzed for concentration of N using dry combustion (CNS2000, LECO Corporation, Saint Joseph, Michigan, USA), and Ca, K, Mg, P, S, Fe, Zn, Mn, Cu, Na, and Al using inductively coupled plasma optical emission spectrometry (ICP Optima 7300 DV, PerkinElmer, Waltham, Massachusetts, USA) at the Department of Soil and Environment, Swedish University of Agricultural Sciences, Uppsala, Sweden. For occasional ears that were malformed, very late maturing, or where the stem had broken, the grains and straw were not analyzed for nutrient content due to the increased risk that retranslocation was affected. The not analyzed material was weighed and the total amount of nutrients in the biomass was calculated by assuming the same concentrations in the damaged as in the undamaged plant parts within the same pot. The yield of minerals, i.e., the weight, was calculated by multiplying the dry weight of biomass by the concentration of the mineral, adjusted for water content. All weights refer to the dry weight. Harvest index (HI) was calculated by dividing the grain biomass by the total aboveground biomass, and N harvest index (NHI) was calculated by dividing the grain N yield by the yield of N in the aboveground biomass.

### Statistics

The SAS version 9.3 procedure Mixed [27] was used for analyzing the variables, with variety as fixed effect and block as random effect. The varieties of different alleles were compared with contrasts to test for significant differences. Only accessions of *Triticum aestivum* ssp *aestivum* L. were included in the comparison of allele types. Normality and heterogeneity of residuals were examined by residual plots, and some variables were transformed to meet the assumptions of the test. The varieties were released during a long period of time and there is a possibility that gradual genetic changes have taken place during this time, which could interfere with the analysis of the comparison of allele types. Therefore, the SAS procedure Reg was used to test for gradual changes in the varieties with time, by performing linear regressions with year of release as the independent variable. All landraces (from NordGen and from the farmer) were treated as if they had been released in 1890.

During the experiment, we observed and graded some leaf spots on many plants, but no relationships to occurrence of any pests/pathogens or nutrient deficiencies were found. To test for the possible effects of the leaf spots on the results of this experiment, the leaf spot grading data were subjected to statistical analysis. The SAS procedure Glimmix with the Cumulative Logit Link function was used to analyze the multinomial variable of the grading, and it was found that the spots were not connected either to allele type or variety. Furthermore, analysis of the variables was repeated with only the most common level of spots, and the results did not change in the majority of cases. Therefore, we did not consider the spots as a factor that affected the comparison of the allele groups in this study.

**Figure 1:**
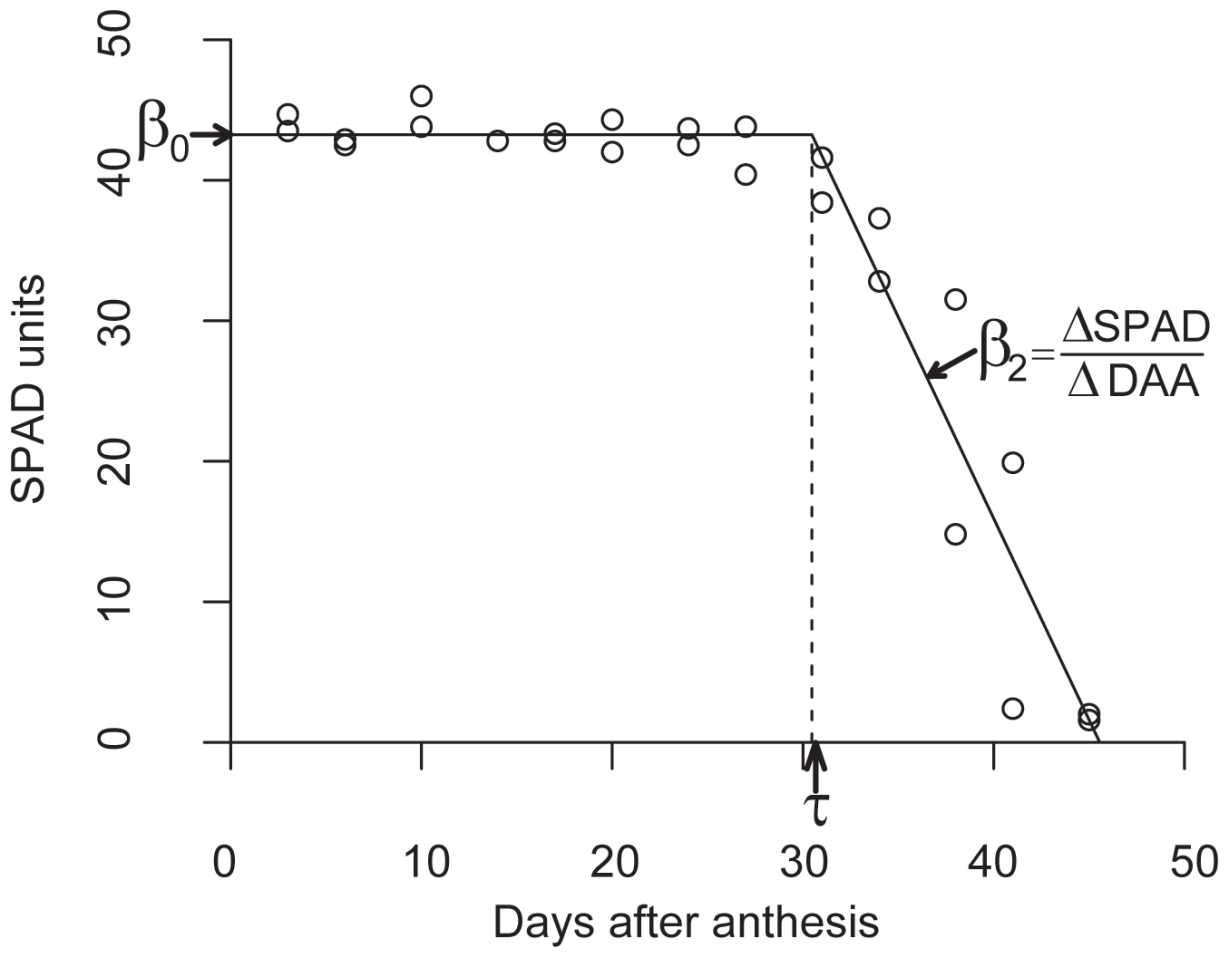
Example of model fit of senescence, variety ‘Vårpärl’ (NGB6675). Parameters are indicated in the graph; β_0_ is the intercept on the y-axis, τ is the breakpoint on the x-axis and β_2_ is the slope of the right segment.

Senescence was analyzed by fitting curves to the SPAD data for each pot, and retrieved values were used in the SAS procedure Mixed model described above. Our different varieties had different curves, and it was not possible to fit one smooth curve to all pots. Therefore, we chose a model which could be fitted to most pots to identify interesting features of senescence, but not always with as perfect a fit as could have been achieved for a few of the pots if a smoother curve had been used. A two-segmented model with a left plateau [28] was fitted for each of the pots, merging the data series for both plants:
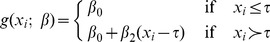
(1)where β_0_ represents the intercept and level of the left segment/plateau, β_2_ is the slope of the right segment which is the senescence rate, and τ is the change point where the two segments meet in this continuous model (Figure 1). In this study, g = SPAD unit and x = days after anthesis. The SAS version 9.2 [27] procedure Nlin with the Marquardt minimization algorithm was used to fit the model. The value of τ was retrieved as the value at start of senescence, and the length of senescence was calculated as the difference in time between τ and the time of completely senesced flag leaf of the slowest plant in each pot. The statistical programming language R version 2.14.2 [29] was used to produce the plots.

## Results

### Leaf Senescence and Development

**Figure 2:**
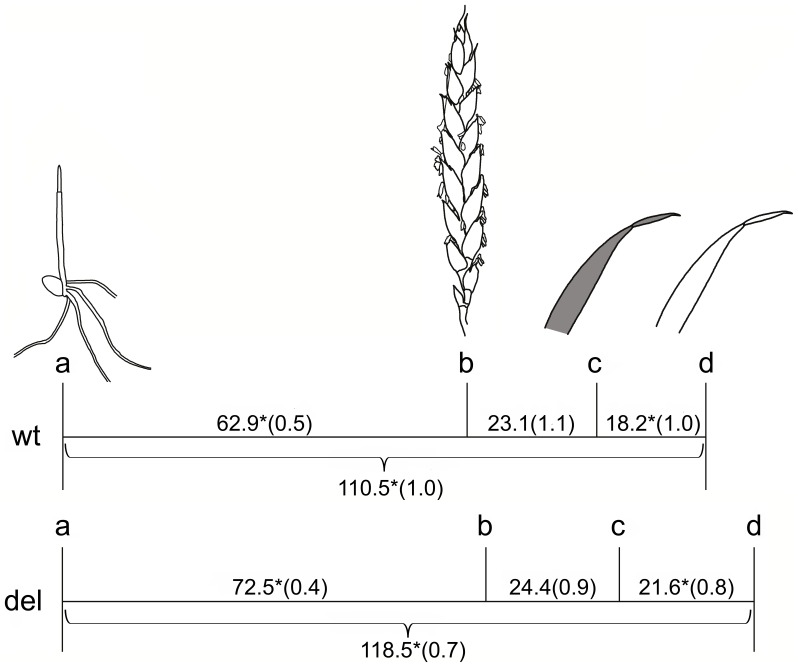
Timeline of development of varieties with the wt allele and varieties with the deletion. The scale is mean number of days (SE in brackets). Time spans that are significantly different between the allele types are indicated with * on both groups of varieties (p<0.001). a = emergence, b = anthesis, c = modeled start of senescence of flag leaf (τ), d = complete senescence of flag leaf.

Varieties with the wt allele completed senescence faster than varieties with the del allele (p = 0.0004) (Figure 2). The range of mean values of the varieties with each type of allele was 11.8–27.3 days for the wt varieties and 15.0–47.0 days for the del varieties. The period between emergence and anthesis was also shorter in the wt varieties (p<0.0001). The total time from emergence to complete senescence of the flag leaf was shorter for the wt varieties than for the del varieties (p<0.0001). There was no difference in the time between anthesis and start of senescence (p = 0.84).

### Biomass

Varieties with the wt allele had significantly lighter grains than varieties with the del allele (Table 2), ranging from 43.9 to 55.6 mg grain^−1^ among the wt varieties and from 39.4 to 57.1 mg grain^−1^ among the del varieties. However, the wt varieties had a larger total grain yield. The wt varieties also had more ears than the del varieties, but there was no difference in the number of grains per ear. The wt varieties had a larger total aboveground biomass than the del varieties, but the del varieties had a higher harvest index. The wt varieties were also taller than the del varieties.

**Table 2:** Means of some agronomic traits for the varieties grouped based on *NAM-B1* allele.

Variable	*NAM-B1* allele						F-value*	p-value*	Emmer mean value**
	wildtype			deletion					
	N	Mean	SE	N	Mean	SE			
Grain yield (g pot^−1^)	44	23.3	1.1	116	20.9	0.6	4.3	0.04	12.8
Weight per grain (mg grain^−1^)	44	48.9	0.6	116	51.2	0.4	20.6	<.0001	34
No. of ears	44	11.4	0.4	114	9.9	0.3	10.1	0.002	11.8
No. of grains per ear	44	40.0	1.6	114	39.2	1.2	0.4	0.56	30.9
Aboveground biomass (g)	44	55.5	2.6	116	47.1	1.3	10.9	0.001	46.3
Plant height (cm)	44	143	2	116	134	2	66.4	<.0001	157
HI (%)	44	42	0.8	116	44	0.5	44	<.0001	28

* F and p-values of contrasts from ANOVA tests with contrasts comparing the wt and del varieties.

** Mean value of the studied emmer wheat (with wildtype *NAM-B1* allele) included for comparison, but was not included in the statistical comparison of allele types.

### Nutrient Content

There were significant differences between the groups of varieties with the different alleles in terms of both macro- and micronutrients in the grain (Table 3). The groups differed in grain macronutrient concentrations of Ca, Mg, P, and S. Of these, the wt group had a higher percentage of Mg and P, and a lower percentage of Ca and S. The yield of the macronutrients Mg and P was significantly higher in the wt group, but there were no significant differences between the groups in yield of the other macronutrients, including N. Regarding the micronutrient concentrations analyzed in the grains, there was only a significant difference in Mn, where the wt group had a lower concentration. There were no differences between the allele groups in the yield of any of the micronutrients in the grain.

**Table 3:** Means of concentrations and total amount per pot of some minerals in the grains for the varieties grouped based on *NAM-B1* allele.

Variable	*NAM-B1* allele						F-value*	p-value*	Emmer mean value**
	wildtype			deletion					
	N	Mean	SE	N	Mean	SE			
N conc. (g g^−1^)	44	0.0213	0.0003	116	0.0218	0.0002	2.5	0.12	2.37
N yield (g pot^−1^)	44	0.489	0.023	116	0.455	0.014	1.8	0.18	0.304
NHI (%)	20	84.8	1.1	28	84.8	0.5	0.0	0.95	–
K conc. (mg kg^−1^)	44	4661	61	116	4656	38	0.0	0.92	5517
K yield (g pot^−1^)	44	0.109	0.006	116	0.098	0.003	3.7	0.06	0.071
Ca conc. (mg kg^−1^)	44	257	6	116	278	4	18.3	<.0001	286
Ca yield (mg pot^−1^)	44	6.04	0.35	116	5.78	0.19	0.4	0.43	3.66
Mg conc. (mg kg^−1^)	44	1412	25	116	1371	12	4.3	0.04	1385
Mg yield (mg pot^−1^)	44	32.6	1.6	116	28.6	0.9	6.3	0.01	17.6
P conc. (mg kg^−1^)	44	3966	53	116	3810	31	9.7	0.002	4681
P yield (mg pot^−1^)	44	92.3	4.8	116	79.6	2.4	7.6	0.007	59.8
S conc. (mg kg^−1^)	44	1573	27	116	1642	13	9.7	0.002	1800
S yield (mg pot^−1^)	44	36.1	1.7	116	34.0	1.0	1.5	0.23	22.9
Fe conc. (mg kg^−1^)	44	32.2	1.2	116	33.3	0.6	1.7	0.19	43.5
Fe yield (mg pot^−1^)	44	0.719	0.033	116	0.675	0.017	2.2	0.14	0.548
Mn conc. (mg kg^−1^)	44	109	2	116	116	1	10.8	0.001	115
Mn yield (g pot^−1^)	44	2.49	0.11	116	2.37	0.06	1.4	0.23	1.46
Zn conc. (mg kg^−1^)	44	40.7	1.6	116	43.2	0.9	3.3	0.07	54.9
Zn yield (mg pot^−1^)	44	0.895	0.035	116	0.870	0.021	0.5	0.49	0.688
Cu conc. (mg kg^−1^)	44	2.7	0.1	116	2.6	0.1	1.2	0.28	2.42
Cu yield (mg pot^−1^)	44	0.064	0.005	116	0.056	0.003	3.2	0.07	0.0305
Al conc. (mg kg^−1^)	44	0.6	0.1	116	0.8	0.1	1.1	0.31	0.491
Al yield (mg pot^−1^)	44	0.015	0.002	116	0.016	0.001	0.1	0.82	0.00622
Na conc. (mg kg^−1^)	44	8.0	0.5	116	9.6	0.4	1.1	0.31	13
Na yield (mg pot^−1^)	44	0.19	0.02	116	0.21	0.01	0.1	0.82	0.169

* F and p-values of contrasts from ANOVA tests with contrasts comparing the wt and del varieties.

** Mean value of the studied emmer wheat (with wildtype *NAM-B1* allele) included for comparison, but was not included in the statistical comparison of allele types.

For the straw, there were significant differences in macronutrient concentrations in the subset of 12 varieties (Table 4). The wt group had lower concentrations of N, K, Ca, Mg, and S. There were also lower yields of the macronutrients Ca and S in the wt group. The straw differed in micronutrients between allele groups, e.g., the concentration of Mn was lower in the wt group and the yield of Fe was higher in the wt group than in the del group. The yield of Al was significantly higher in the straw of the wt group. About 85% of the N was in the grain (NHI) in both allele groups.

**Table 4:** Means of concentrations and total amount per pot of some minerals in the straw for a subset of 12 spring wheat varieties, grouped based on *NAM-B1* allele.

Variable	*NAM-B1* allele						F-value*	p-value*
	wildtype			deletion				
	N	Mean	SE	N	Mean	SE		
N conc. (g kg^−1^)	20	2.80	0.18	28	3.24	0.10	7.3	0.01
N yield (g pot^−1^)	20	0.0764	0.0067	28	0.0835	0.0051	1.0	0.33
K conc. (mg kg^−1^)	20	14141	448	28	15635	450	8.7	0.006
K yield (g pot^−1^)	20	0.391	0.032	28	0.406	0.025	0.2	0.64
Ca conc. (mg kg^−1^)	20	1718	78	28	2366	148	27.1	<.0001
Ca yield (mg pot^−1^)	20	48.9	5.5	28	61.9	4.9	5.5	0.03
Mg conc. (mg kg^−1^)	20	734	35	28	925	50	14.6	0.0006
Mg yield (mg pot^−1^)	20	20.9	2.3	28	24.3	1.8	2.6	0.12
P conc. (mg kg^−1^)	20	1477	98	28	1506	72	0.1	0.78
P yield (mg pot^−1^)	20	42.8	4.9	28	40.0	3.1	0.5	0.50
S conc. (mg kg^−1^)	20	2336	154	28	2979	133	16.4	0.0003
S yield (mg pot^−1^)	20	59.7	2.4	28	73.9	3.2	14.5	0.0006
Fe conc. (mg kg^−1^)	20	19.7	2.3	28	16.0	1.6	2.8	0.11
Fe yield (mg pot^−1^)	20	0.565	0.100	28	0.375	0.030	5.3	0.03
Mn conc. (mg kg^−1^)	20	273	13	28	312	15	5.4	0.03
Mn yield (mg pot^−1^)	20	7.21	0.41	28	7.80	0.39	2.1	0.15
Zn conc. (mg kg^−1^)	20	14.7	1.2	28	14.4	0.7	0.1	0.78
Zn yield (mg pot^−1^)	20	0.400	0.035	28	0.366	0.022	1.7	0.20
Cu conc. (mg kg^−1^)	20	1.4	0.1	28	1.6	0.1	1.7	0.20
Cu yield (mg pot^−1^)	20	0.042	0.005	28	0.041	0.003	0.0	0.97
Al conc. (mg kg^−1^)	20	2.4	0.2	28	2.2	0.2	1.3	0.27
Al yield (mg pot^−1^)	20	0.064	0.005	28	0.054	0.003	6.0	0.02
Na conc. (mg kg^−1^)	20	41.8	4.7	28	27.8	1.6	15.5	0.0004
Na yield (mg pot^−1^)	20	1.26	0.22	28	0.73	0.06	11.7	0.002

* F and p-values of contrasts from ANOVA tests with contrasts comparing the wt and del varieties.

### Gradual Changes with Time

**Figure 3:**
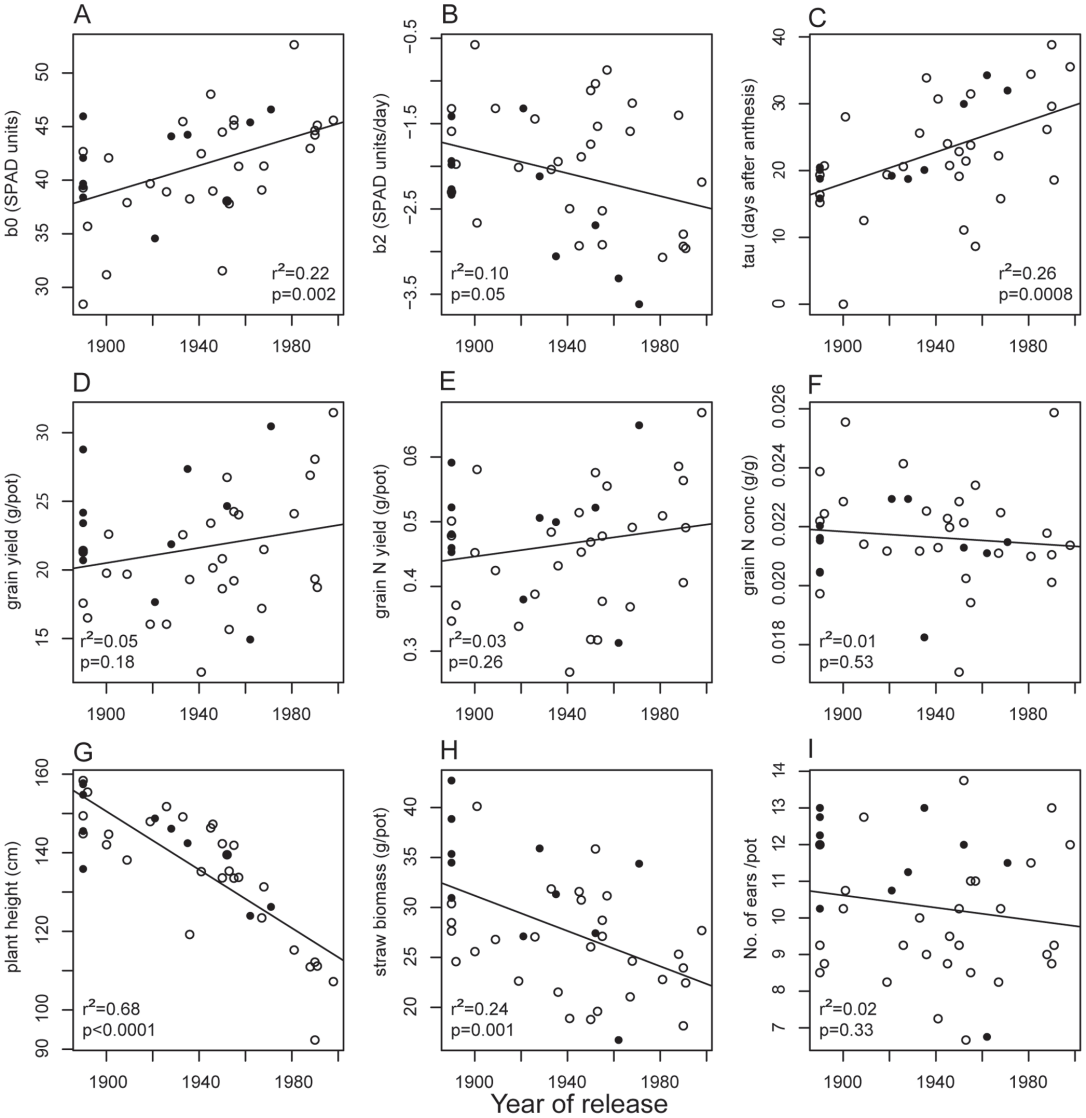
Changes in measured traits over time. Filled circles are varieties with the wildtype allele of *NAM-B1*, open circles are varieties with the deletion. A) modeled SPAD-units at anthesis, β_0_, B) rate of senescence, β_2_, C) modeled start of senescence in days after anthesis, τ, D) grain yield, E) grain N yield, F) grain N concentration, G) plant height, H) straw biomass, I) number of ears per pot.

**Figure 4:**
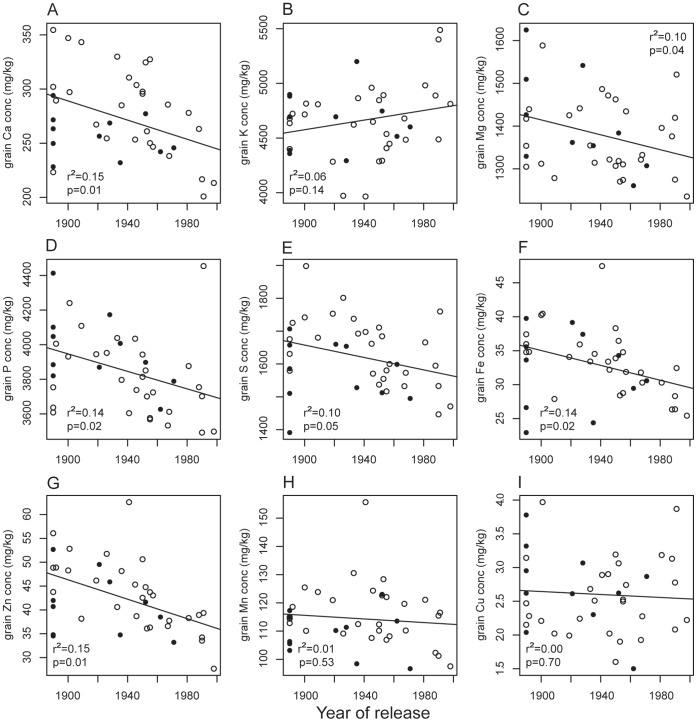
Changes in grain mineral concentrations over time. Filled circles are varieties with the wildtype allele of *NAM-B1*, open circles are varieties with the deletion. The graph indicates grain concentrations of A) calcium, B) potassium, C) magnesium, D) phosphorus, E) sulfur, F) iron, G) zinc, H) manganese, and I) copper.

Grain yield did not change significantly with release year of the variety, but straw biomass decreased (Figure 3). The del varieties had significantly lower straw biomass, which made it difficult to separate the effect of gradual genetic changes from the effect of the specific *NAM-B1* allele in this trait. The absence of trend in grain biomass, on the other hand, supported the finding of a significant difference between the alleles in grain biomass. The plants became significantly shorter with release year, and again the del varieties had shorter straw than the wt varieties. The grain N concentration and N yield did not change significantly with release year, and neither of these traits differed between the allele groups. The concentration of several other grain minerals decreased (Figure 4), but since yield increased (even if not significantly), there was no significant trend in total yield for any of the minerals tested (data not shown). The modeled senescence parameters changed with release year: SPAD value at anthesis increased (with no difference between allele groups), the senescence rate was faster (wt varieties had faster senescence and these results are therefore supported), and senescence started later with release year (there was no significant difference in start of senescence between allele groups) (Figure 3). Although several of the variables changed significantly with year of release, the regressions still only explained relatively small parts of the variation in the variables based on the coefficients of determination (r^2^). Plant height had the highest value (0.68), and we considered that regression to be strong. The other regressions had much lower r^2^ values.

## Discussion

Varieties with the wt allele of *NAM-B1* had faster development and completed leaf senescence faster. They also had lighter grains and higher yield of P and K in the grain compared with varieties with the del allele. In our set of 40 Swedish spring wheat varieties, the pleiotropic effects of *NAM-B1* genotype appeared consistent and large enough to be detectable when plants with different genetic backgrounds were compared.

To increase precision, we chose to study the effect of a single gene (*NAM-B1*) on a number of phenotypic traits under controlled conditions, in which additional variation caused by the actions of pests and diseases and other environmental factors was minimized. The values of yield and other traits assessed in controlled greenhouse environments frequently differ from results obtained in the field. For example, the number of ears per plant was much greater in our study than in the field. Therefore, field experiments could give additional information in evaluating the effect of the gene on e.g., yield components and nutrient retranslocation. However, we believe that the results to a large extent reflect the relative differences between the varieties. When two of the varieties, a landrace from Dalarna obtained from a farmer and Vinjett, were recently grown together in a field experiment at about 180 kg N ha^−1^, the grain N concentrations were similar to those in the present study (2.1% and 1.9% for Vinjett in climate chamber vs field, 2.2% in both experiments for the Dalarna landrace; unpublished data), indicating that there is agreement between this climate chamber study and the field situation.

A relevant question is whether differences in phenotype between the varieties carrying different alleles are influenced by other genes than *NAM-B1*. A more precise method would be to compare the phenotype of NILs carrying different *NAM-B1* alleles in different Swedish variety backgrounds. Another approach would be to reduce the transcript level of the wt allele in different Swedish varieties [10] and study the resulting phenotype. Such lines are not currently available. However, our study exemplifies an approach of investigating effects of single alleles which can be used when NILS are not available in a given genetic background. The fact that many of the results in our study are in line with previously shown effects of the wt *NAM-B1* allele makes it likely that some of the phenotypic differences between the two variety groups studied are actually controlled by *NAM-B1*. The potential pleiotropic effect of the wt allele in different genetic backgrounds is of great interest in developing new bread wheat varieties with qualities controlled by the wt *NAM-B1* allele.

In parallel to historical developments in which the presence of the wt allele decreased during the 20^th^ century in Fennoscandian varieties [15], some traits may have changed due to directional breeding. This poses a problem of separating the effects of the allele from the effects of other genes on the same traits. That is, the allele type may be causing a trend in a trait over years, or a trend can lead us to see a difference between allele types in the traits. The difference in plant height between the allele groups, for example, with the wt allele varieties being higher, could possibly be explained by directional breeding. Breeding has targeted shorter varieties, but there is no known effect of the wt allele on plant height. However, there are too few varieties with and without the wt allele originating from the same year to allow for a study without such trends. The study performed here can at least give an indication of the effect of the wt allele in these Swedish varieties, which are unique from an international perspective. The absence or opposite direction of gradual changes in many traits believed to be affected by *NAM-B1*, e.g., grain N concentration and senescence rate, supports our results for these traits.

Breeding has made a positive contribution to bread wheat yields in many countries during the 20^th^ century [30], and a yield increase has been reported for Nordic spring wheat varieties under field conditions, even if the r^2^ was low in that study [31]. We did not find a significant yield increase with year of release in this study of spring wheat. Furthermore, we did not find a reduction in grain N concentration with time, as has been found in Argentinian [32], [33] and Italian [34] bread wheat cultivars. It is known that there is often a negative relationship between grain yield and grain N concentration [35], [36]. If previous breeding aimed for high N concentrations, it is possible that yield increases have been low as a consequence. The fact that the varieties were grown close together in this study may also have given an advantage to the higher (and in general older) varieties.

A novel finding was that varieties with the wt allele reached anthesis faster than the other varieties, which has not been observed previously for wheat. However, it has been noticed in a high grain protein concentration barley NIL [37]. Barley carries an ortholog of *NAM-B1* called *HvNAM-1*, which also has effects on senescence and grain protein concentration [37]–[39]. Wheat is sensitive to heat spells and droughts around anthesis, which reduce the number of grains per ear [40]. Earlier anthesis is one way to escape this drought risk [41]. Fast maturation can be a desirable trait in cases of adverse conditions at the end of the growing season (e.g., cold and rain), which are common in the Fennoscandian climate. After the wt allele had been identified in Swedish germplasm, it was hypothesized that the faster maturation of the wt allele had led to its preservation also in other areas at high latitudes [15]. However, there was little evidence supporting this hypothesis, since the wt allele was found only in Fennoscandian varieties, and not in varieties grown in other areas at high latitude [15]. Nevertheless, if the wt varieties have faster maturation without a reduction in yield and possibly even a yield increase, this may still be one reason explaining why the wt allele has been retained in Fennoscandia. In the early Swedish breeding programs landraces were used in crosses with the deliberate goal of transferring their early ripening [42]. Some of the landraces used in the crosses (originating in Dalarna and Halland, Table 1) most likely carried the wt allele.

Varieties with the wt allele had lighter grains than varieties with the non-functional allele type, as hypothesized. However, they had higher total grain weight and more ears, which has not previously been reported as effects of the wt allele. In a study of the effect of the gene in some Indian bread wheat varieties into which the wt allele had been crossed, the lines with the wt allele all had more tillers than the lines from which they were derived [13]. This may be an actual effect of the wt allele that is only expressed in certain varieties or environments. It is also possible that the wt allele is linked to, or controlled by, other genes in the Swedish varieties studied here. More ears is a way of increasing the number of grains per unit area [43] and could be a way of increasing yield at a fixed N fertilization rate.

The concentrations and yields of N, Fe, Zn, and Mn in our Swedish varieties with the wt *NAM-B1* allele were similar to those of varieties with a non-functional allele, contrary to our expectations based on previous reports of *NAM-B1* effects on nutrient retranslocation [10], [14], [16]–[18]. This indicates that possible effects of the wt allele on N use efficiency through increased grain N concentration [2] are probably negligible. The only difference in yield was in the macronutrients P and Mg. It is possible that differences in micronutrients were too small to detect. Previous results indicate that the difference in minerals between allele types is smaller closer to complete senescence [18], and we harvested when all plants were mature, which would have decreased the differences.

An observation relevant for breeding is that we did not find indications of reduced yield in wt varieties in the environment studied here. Instead, the yield was higher in the wt varieties. This is positive if the allele is to be used in breeding, even if the effect on yield is known to be different in different environments. The absence of differences between the variety groups in grain N concentration and grain N yield indicates that any effects of the wt allele on these traits are small in some conditions, a fact which needs to be considered if the allele is to be used for increasing grain N. Since the varieties with the wt allele had lower amounts of several micro- and macronutrients in the straw, the question of retranslocation remains unclear. This issue is probably better examined by more detailed studies of nutrient allocation in NILs. Since varieties with the wt allele did not have higher grain N concentration or grain N yield, we have no indications that the good baking properties of Swedish spring wheat are related to the wt *NAM-B1* allele. However, it would be interesting to study this aspect under field conditions. Varieties with the wt allele reached anthesis faster and senesced faster than varieties with a non-functional allele, which is also interesting for breeding purposes.

### Conclusions

Two groups of varieties, with the wt or the del allele of *NAM-B1*, differed in several traits. Varieties with the wt allele had lighter grains, more ears, and reached anthesis and completed senescence faster. The wt varieties had higher concentrations of Mg and P in the grain, while the del varieties had higher concentrations of Ca, S, and Mn in the grain. These findings suggest that the wt *NAM-B1* allele has multiple effects on the phenotype that are detectable when the different alleles are present in different genetic backgrounds. No difference between variety groups was found in grain N concentration or grain N yield, which does not support an association between the *NAM-B1* wt allele and the high baking quality of Swedish spring wheat. It also indicates that the relationship between nitrogen use efficiency and allele type might be small. The higher number of ears and faster development and senescence in *NAM-B1* wt varieties is interesting for breeding purposes and should be further investigated.
